# New cheiracanthiid spiders from Xishuangbanna rainforest, southwestern China (Araneae, Cheiracanthiidae)

**DOI:** 10.3897/zookeys.940.51802

**Published:** 2020-06-11

**Authors:** Jianshuang Zhang, Hao Yu, Shuqiang Li

**Affiliations:** 1 School of Life Sciences, Guizhou Normal University, Guiyang, Guizhou, China Guizhou Normal University Guiyang China; 2 School of Biological Sciences, Guizhou Education University, Guiyang, Guizhou, China Guizhou Education University Guiyang China; 3 Institute of Zoology, Chinese Academy of Sciences, Beijing, China Institute of Zoology, Chinese Academy of Sciences Beijing China

**Keywords:** key to genera, new genus, new species, Oriental Region, taxonomy

## Abstract

Four new species of the genus *Cheiracanthium* C.L. Koch, 1839 from Xishuangbanna, Yunnan Province, China are described: *C. daofeng* Yu & Li, **sp. nov.** (♂♀), *C. duanbi* Yu & Li, **sp. nov.** (♂♀), *C. gou* Yu & Li, **sp. nov.** (♂), and *C. wuquan* Yu & Li, **sp. nov.** (♀). In addition, *Sinocanthium* Yu & Li, **gen. nov.**, is described with the type species *S. shuangqiu* Yu & Li, **sp. nov.** A key to cheiracanthiid genera distributed in East and Southeast Asia is provided.

## Introduction

Cheiracanthiidae Wagner, 1887 is a relatively large spider family with twelve genera and 354 valid species distributed worldwide, except for the Polar Regions (WSC 2020). The Cheiracanthiidae fauna of China is presently known by 42 described species, all of them belonging to *Cheiracanthium* C.L. Koch, 1839 sensu lato (Li 2020). The global diversity of this family remains insufficiently studied, and several new species have been described recently (Lotz 2015; Marusik and Fomichev 2016; Zhang et al. 2018; Li and Zhang 2019). The current paper reports new Cheiracanthiidae taxa from Xishuangbanna.

Xishuangbanna, which lies between 21°08'–22°36'N and 99°56'–101°50'E, is in southwestern China and shares a border with Myanmar in the southwest and Laos in the southeast. Xishuangbanna is a biodiversity hotspot, and a large number of new taxa across a wide variety of spider families have been discovered in this region recently (Li 2020). Seven *Cheiracanthium* species were reported from Xishuangbanna before the current study. They are *C. exquestitum* Zhang & Zhu, 1993, *C. falcatum* Chen, Huang, Chen & Wang, 2006, *C. insigne* O. Pickard-Cambridge, 1874, *C. insulanum* (Thorell, 1878), *C. ningmingense* Zhang & Yin, 1999, *C. taiwanicum* Chen, Huang, Chen & Wang, 2006, and *C. unicum* Bösenberg & Strand, 1906.

During the examination of spiders collected from 2006 to the present in Xishuangbanna Tropical Botanical Garden in Menglun Town, four species recognized as new to science are described here and are temporarily placed in *Cheiracanthium* sensu lato. The fifth species is not readily assignable to any of the existing genera; thus, we established a new genus to accommodate it. Detailed morphological descriptions and diagnoses of the new taxa are given. The body and the copulatory organs are photographed and illustrated for each species. An identification key to cheiracanthiid genera occurring in East and Southeast Asia is provided.

## Materials and methods

Specimens were collected by fogging, pitfall trapping, and hand collecting from the canopy, tree trunks, and leaf litter in the tropical rainforest in Xishuangbanna Tropical Botanical Garden and preserved in 75 or 95% ethanol. All type specimens are deposited in the Institute of Zoology, Chinese Academy of Sciences (IZCAS) in Beijing, China (curator: Jun Chen).

Specimens were examined using a LEICA M205C and an Olympus SZX7 stereomicroscope. Further details were studied under a CX41 compound microscope. Male and female copulatory organs were examined and illustrated after dissection. Left male palps are illustrated, unless otherwise indicated (photos of the right palp were horizontally mirrored in the figures to allow easier comparison with other species). Epigynes were removed and cleared in lactic acid or warm 10% potassium hydroxide (KOH) solution. Some vulvae were imaged after being embedded in Arabic gum. Images were captured with a Canon EOS 70D digital camera mounted on an Olympus CX41 compound microscope and assembled using Helicon Focus 6.80 image stacking software. All measurements were obtained using an Olympus SZX7 stereomicroscope and are given in millimetres. Eye diameters are taken from the widest distance. The total body length does not include chelicerae or spinnerets. Leg lengths are given as total length (femur, patella + tibia, metatarsus, tarsus). Most of the terminology in the text and figure legends follows Lotz (2015) and Zhang et al. (2018), and some follows Marusik and Fomichev (2016) and Morano and Bonal (2016). Abbreviations used in the text and figures are as follows:

**A** atrium

**AAM** atrial anterior margin

**AER** anterior eye row

**AL** abdomen length

**ALE** anterior lateral eyes

**AME** anterior median eyes

**AME–AME** distance between AMEs

**AME–ALE** distance between AME and ALE

**APM** atrial posterior margin

**AW** abdomen width

**C** conductor

**CD** copulatory duct

**CF** cymbial fold

**CI** carapace index

**CL** carapace length

**CL/CW** carapace length / carapace width

**CLL** clypeal length

**CO** copulatory opening

**CS** cymbial spur

**CW** carapace width

**DTA** dorsal tibial apophysis

**E** embolus

**EB** embolic base

**FD** fertilisation duct

**LL** total length of leg I

**LL/CL** leg I / carapace length

**MA** median apophysis

**MOQ** median ocular quadrangle

**MOQA**MOQ anterior width

**MOQP**MOQ posterior width

**OAL** ocular area length

**OAW** ocular area width

**PER** posterior eye row

**PLE** posterior lateral eyes

**PME** posterior median eyes

**PME–PME** distance between PMEs

**PME–PLE** distance between PME and PLE

**PMT** promarginal teeth

**PTA** prolateral tibial apophysis

**R** receptacle

**RMT** retromarginal teeth

**RTA** retrolateral tibial apophysis

**STL** sternum length

**STW** sternum width

**TL** total body length

A DNA barcode was also obtained for species delimitation and matching of different sexes. A partial fragment of the mitochondrial cytochrome oxidase subunit I (CO1) gene was amplified and sequenced using the primers LCOI1490 (5’-GGTCAACAAATCATAAAGATATTG-3’) and HCOI2198 (5’-TAAACTTCAGGGTGACCAAAAAAT-3’). For additional information on extraction, amplification, and sequencing procedures, see Morano and Bonal (2016). All sequences were analysed using BLAST and are deposited in GenBank. The accession numbers are provided in Table 1.

**Table 1.** Voucher specimen information.

Species	Voucher code	Sex	GenBank accession number	Sequence length
*C. daofeng* sp. nov.	YHCH010	♂	MT449426	664bp
	YHCH021	♀	MT478102	665bp
*C. duanbi* sp. nov.	YHCH030	♂	MT478103	665bp
	YHCH029	♀	MT478104	665bp
*C. gou* sp. nov.	YHCH016	♂	MT477871	665bp
*C. wuquan* sp. nov.	YHCH020	♀	MT477870	664bp
*S. shuangqiu* sp. nov.	YHCH011	♀	MT478105	665bp

## Taxonomy

### Family Cheiracanthiidae Wagner, 1887

#### Key to cheiracanthiid genera occurring in East and Southeast Asia (females)

**Table:** 

1	Trochanters not notched; epigynal atrium absent or reduced (Deeleman-Reinhold 2001: 251, fig. 335)	*** Calamoneta ***
–	Trochanters notched (Deeleman-Reinhold 2001: 64, fig. 94); epigynal atrium present (Figs 2A, 2B, 4A, 4B, 6C, 6D, 6F, 11C, 11D, 11F; Deeleman-Reinhold 2001: 241, fig. 305; 247, figs 323, 328)	**2**
2	Small-sized spiders (less than 4 mm)	*** Summacanthium ***
–	Medium to large-sized spiders, usually larger than 4 mm (Figs 2G, 2H, 4G, 4H, 6A, 6B, 11A, 11B)	**3**
3	Fragile cheiracanthiids with a slender body and greenish colour (Deeleman-Reinhold 2001: 247, figs 321, 322), carapace flattened (Deeleman-Reinhold 2001: 247, fig. 321), venter of abdomen with colour pattern (Deeleman-Reinhold 2001: 247, fig. 322), trochanters shallowly notched, metatarsus and tarsus I flexible, with pseudosegments	*** Calamopus ***
–	Yellow or brownish spider, sometimes with reddish or dark brown head, carapace not flattened, abdomen ventrally without distinct colour pattern (Figs 2G, H, 4G, H, 6A, B, 11A, B), trochanters are deeply notched, metatarsus and tarsus I inflexible, without pseudosegments	**4**
4	Copulatory ducts absent; atrium located anteriorly, with delimited margin posteriorly (Fig. 11C–G)	***Sinocanthium* gen. nov.**
–	Copulatory ducts distinct, with variable shapes, lengths and courses; atrium located posteriorly or centrally, usually rebordered anteriorly and laterally (Figs 2A–D, 4A–D, 6C–G)	***Cheiracanthium* sensu lato**

#### Key to cheiracanthiid genera occurring in East and Southeast Asia (males)

**Table:** 

1	Trochanters not notched; cymbium without spur (Deeleman-Reinhold 2001: 251, figs 333, 334)	*** Calamoneta ***
–	Trochanters notched (Deeleman-Reinhold 2001:64, fig. 94); cymbial spur present (Figs 7–9; Deeleman-Reinhold 2001: 241, figs 303, 304; 243, figs 310, 311; 246, figs 315, 316; 247, fig. 327)	**2**
2	Small-sized spiders (less than 4 mm); abdomen ventrally with a scape-shaped bulge, bulge laterally with chitinized pits (Deeleman-Reinhold 2001: 241, figs 301, 302; 243, figs 307, 308)	*** Summacanthium ***
–	Medium- to large-sized spiders, usually larger than 4 mm; abdomen unmodified (Figs 2E, 2F, 4E, 4F)	**3**
3	Fragile cheiracanthiids with a slender body and greenish colour (Deeleman-Reinhold 2001: 246, fig. 312; 247, figs 319, 320), carapace flattened (Deeleman-Reinhold 2001: 247, fig. 318), venter of abdomen with colour pattern (Deeleman-Reinhold 2001: 247, fig. 320), trochanters notched shallowly, metatarsus and tarsus I flexible with pseudosegments; cymbial spur reflexed near the base, directed alongside the dorsal cymbium, directed apically alongside the dorsal cymbium (Deeleman-Reinhold 2001: 246, figs 315, 316; 247, 327)	*** Calamopus ***
–	Yellow or brownish spider, sometimes with reddish or dark brown head, carapace not flattened, abdomen ventrally without distinct colour pattern (Figs 2E, F, 4E, F, 5D–F), trochanters are deeply notched, metatarsus and tarsus I inflexible without pseudosegments; cymbial spur not flexed at base, pointing in variable directions	***Cheiracanthium* sensu lato**

**Comments.** The debate on the group’s limits and internal structure of this family remains open (Deeleman-Reinhold 2001), and there is much dispute about the family placement of Neotropical genera (i.e. *Eutichurus* Simon, 1897) (Marusik and Fomichev 2016). Within Cheiracanthiidae, four genera from East and Southeast Asia can be considered most closely related to the type genus *Cheiracanthium*.

#### Genus Cheiracanthium

Taxon classificationAnimaliaAraneaeMiturgidae

C.L. Koch, 1839

527BA05F-4CE8-5131-B223-9939FD9DC474

Chiracanthops Mello-Leitão 1942 (Cheiracanthium is considered a senior synonym of Chiracanthops, Bonaldo and Brescovit 1992: 732).
Cheiracanthium C.L. Koch 1839: 9 (type species: Aranea punctoria Villers, 1789); Simon 1897: 87; Simon 1932: 895; Petrunkevitch 1933: 53; Reimoser 1937: 71; Edwards 1958: 368; Lehtinen 1967: 291; Dondale and Redner 1982: 17; Roberts 1985: 88; Sterghiu 1985: 100; Yaginuma 1986: 177; Chikuni 1989: 122; Paik 1990: 3; Wolf 1991: 233; Bonaldo and Brescovit 1992: 731; Deeleman-Reinhold 2001: 224; Lotz 2007a: 4; 2007b: 148; 2011: 23; 2014: 303; 2015: 322; Li and Lin 2016: 78.
Helebiona Benoit, 1977: 80 (type species: H. wilma Benoit, 1977); Lotz 2007a: 66.

##### Type species.

*Aranea punctoria* Villers, 1789, type locality: Europe.

##### Comments.

The genus *Cheiracanthium* currently includes 215 extant species that are widespread in the Old World and represent 61% of the total number of Cheiracanthiidae species (WSC 2020). However, the genus remains inadequately studied because: (1) almost half of the species are known from a single sex or juveniles (37 from males, 59 from females, two from juveniles), and in some cases, the adults are apparently mismatched, or conspecific male and females have been described as separate species (Dankittipakul and Beccaloni 2012; WSC 2020); (2) original descriptions are rather brief and lack illustrations or the illustrations are inadequate (Zhang et al. 2018); (3) the diversity of this genus is still insufficiently known (Zhang et al. 2018). Although several major taxonomic studies on a regional scale have been conducted, e.g., Edwards (1958) for the US, Wolf (1991) for Central Europe, Bonaldo and Brescovit (1992) for the Neotropical Region, Lotz (2007a, b, 2011, 2014, 2015) for the Afrotropical Region, Deeleman-Reinhold (2001) for Southeast Asia, the genus *Cheiracanthium* has been widely considered paraphyletic (Wunderlich 2012; Marusik and Fomichev 2016). We agree with Bayer (2014) regarding the need of an extensive, large-scale review of the genus. Consequently, the present study follows the WSC (2020) and temporarily places the four new species in *Cheiracanthium* sensu lato.

#### Cheiracanthium daofeng

Taxon classificationAnimaliaAraneaeMiturgidae

Yu & Li sp. nov.

6E4DEEFB-50A8-5442-91F9-BC3F05955C22

http://zoobank.org/148233AF-5A6E-42EC-B4ED-B3E7258631DD

**Figure 1.**
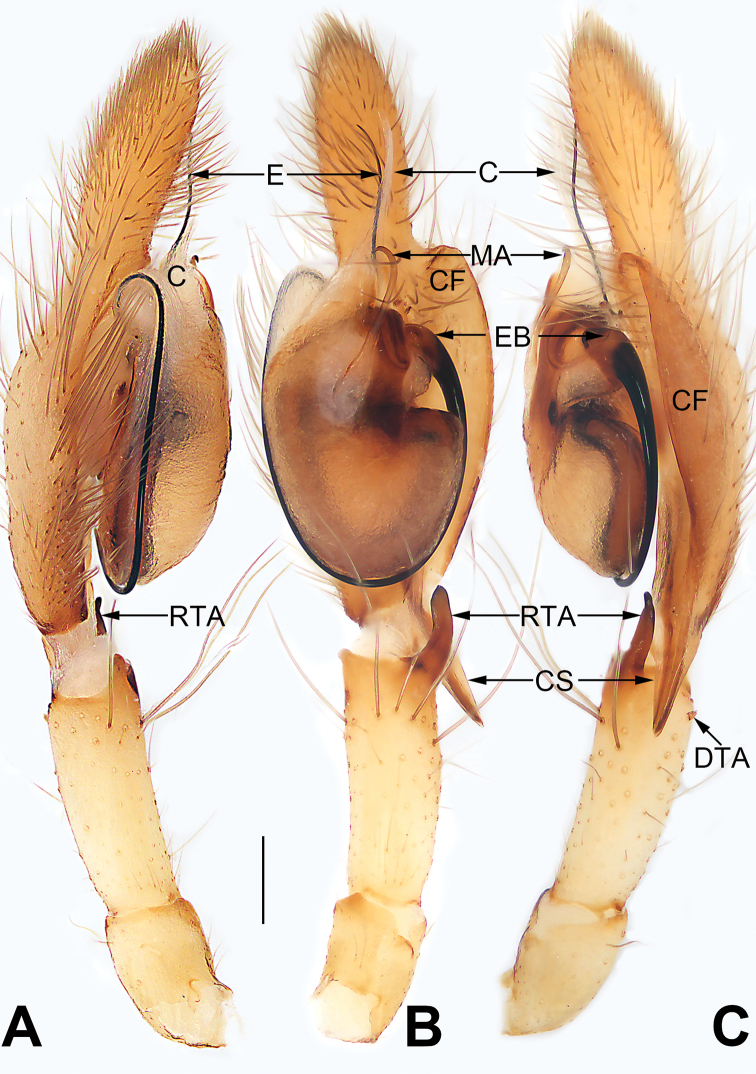
Palp of *Cheiracanthium daofeng* sp. nov., male holotype. **A** Prolateral view **B** ventral view **C** retrolateral view. Abbreviations: C = conductor; CF = cymbial fold; CS = cymbial spur; DTA = dorsal tibial apophysis; E = embolus; EB = embolic base; MA = median apophysis; RTA = retrolateral tibial apophysis. Scale bar: 0.2 mm.

**Figure 2.**
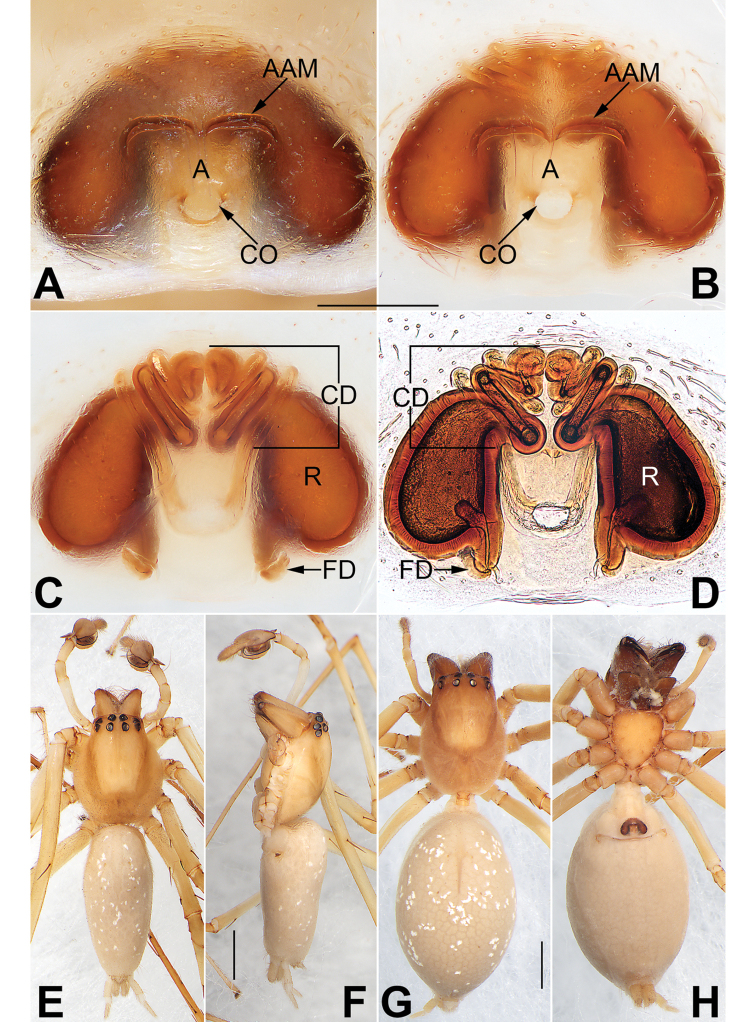
*Cheiracanthium daofeng* sp. nov., female paratype and male holotype. **A** Epigyne, intact, ventral view **B** epigyne, cleared, ventral view **C** vulva, cleared, dorsal view **D** vulva, cleared and embedded in Arabic gum, dorsal view **E** male habitus, dorsal view **F** male habitus, lateral view **G** female habitus, dorsal view **H** female habitus, ventral view. Abbreviations: A = atrium; AAM = atrial anterior margin; CD = copulatory duct; CO = copulatory opening; FD = fertilization duct; R = receptacle. Scale bars: 0.2 mm (**A–D**); 1 mm (**E–H**).

Figures 1 , 2 , 7A , 8A , 9A , 10A–C

##### Holotype.

♂ (IZCAS-Ar 34741), China, Yunnan Province, Xishuangbanna, Mengla County, Menglun Town, Xishuangbanna Tropical Botanical Garden, seasonal tropical primary rainforest, 21°54.725'N, 101°13.261'E, elevation ca. 734 m, 8.VIII.2007, Guo Zheng leg. Paratype: 1♀ (IZCAS-Ar 34742), same data as holotype.

##### Other material examined.

China, Yunnan Province, Xishuangbanna, Mengla County, Menglun Town, 1♂ (YHCH010), Baka Village, 21°57.703'N, 101°12.062'E, elevation ca. 736 m, 5.VIII.2012, Guo Zheng leg.; 1♀ (YHCH021), Bubang Village, monsoon forest, 21°36.827'N, 101°34.847'E, elevation ca. 690 m, 12.VIII.2012, Guo Zheng leg.

##### Etymology.

The specific name is derived from the Chinese pinyin ‘dāo fēng’, which means ‘blade point’, referring to the narrow, sharp conductor which is shaped like a blade; noun in apposition.

##### Diagnosis.

**Figure 3.**
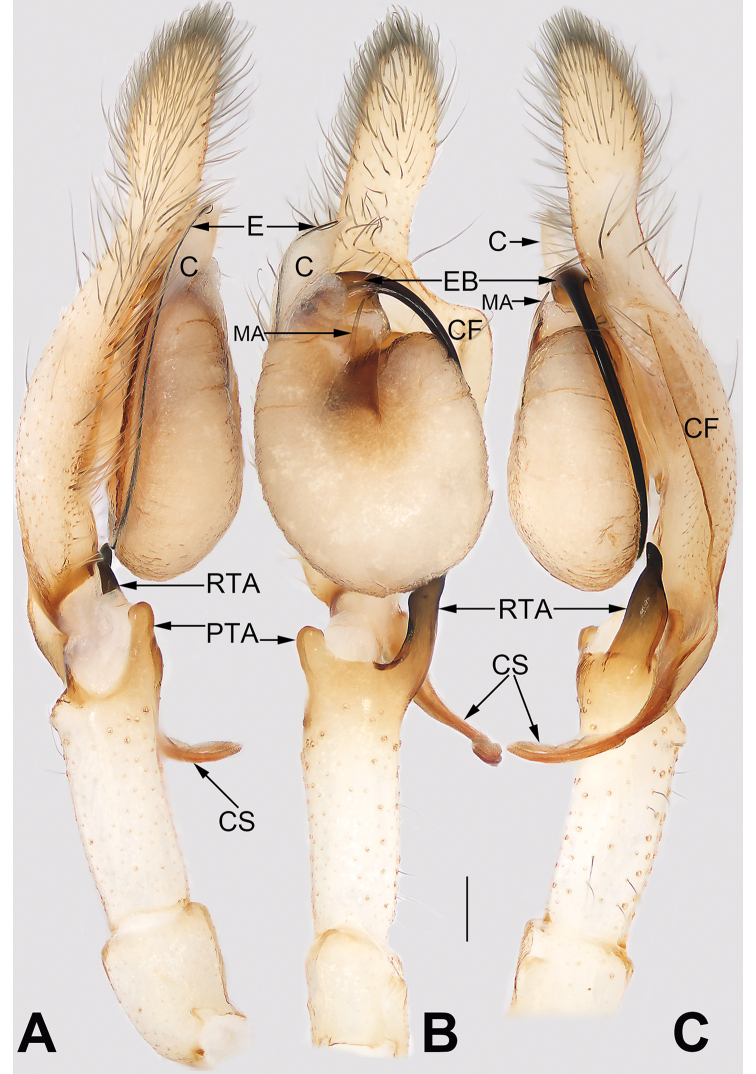
Palp of *Cheiracanthium daofeng* sp. nov., male holotype. **A** Prolateral view **B** ventral view **C** retrolateral view. Abbreviations: C = conductor; CF = cymbial fold; CS = cymbial spur; E = embolus; EB = embolic base; MA = median apophysis; PTA = prolateral apophysis; RTA = retrolateral tibial apophysis. Scale bars: 0.2 mm.

Males of the new species can be easily distinguished from the congeners by the blade-shaped pointed conductor with a sharp tip and by the embolar tip extending to the apex of the cymbium (Figs 1A–C, 7A, 8A, 10A–C) (the conductor and the embolar tip are relatively short and do not extend to the cymbial tip in almost all other *Cheiracanthium* species, such as *C. duanbi* sp. nov. and *C. gou* sp. nov.; Figs 3A–C, 5A–C, 7B, C, 8B, C, 10D–I). The females are similar to those of *C. taiwanicum* (Chen and Huang 2012: 25, fig. 7E, F) in having a similar habitus, an eyebrow-shaped atrial anterior margin, and coiled copulatory ducts but differ in the following: (1) the receptacles are kidney-shaped (Fig. 2C, D) (vs. globular in *C. taiwanicum*), and (2) having different numbers of copulatory duct coils (four coils in *C. daofeng* sp. nov., vs. six coils in *C. taiwanicum*) (Fig. 2C, D).

##### Description.

**Male.** Holotype (Figs 2E, F): TL 4.95; CL 2.15, CW 1.74, CI (CL/CW) 1.24; AL 2.80, AW 1.34. Carapace pale brown, with pair of brown lateral bands extending from behind PME and PLE, almost reaching posterior margin, fused, forming a U-shaped patch. Eyes: AER slightly recurved, PER slightly wider than AER, almost straight in dorsal view. All eyes dark with black rings. Eye sizes and interdistances: OAL 0.37, OAW 0.99; AME 0.12, ALE 0.14, PME 0.11, PLE 0.13; AME–AME 0.07, AME–ALE 0.10, PME–PME 0.17, PME–PLE 0.27; MOQA 0.37, MOQP 0.45, CLL 0.05. Chelicerae protruding and coloured as carapace, with a small basal condyle, three teeth on promargin and three on retromargin. Sternum pale brown, STL 1.04, STW 0.91. Labium and endites coloured as carapace. Legs pale yellow, without distinct markings. Leg measurements: I 21.08 (5.36, 6.70, 6.72, 2.30), II 11.50 (3.11, 3.83, 3.66, 0.91), III 8.58 (2.35, 2.57, 2.85, 0.82), IV 12.28 (3.24 3.76, 4.27, 1.02); LL/CL 9.80. Abdomen lanceolate, dorsally yellowish white, dorsum with indistinct heart-shaped mark, two pairs of inconspicuous muscle depressions, scattered numerous white pigmented spots; venter yellowish white without distinct pattern.

Palp (Figs 1A–C, 7A, 8A, 9A, 10A–C). Tibia with two apophyses: a retrolateral one that is relatively long and sclerotized, ca. 1/3 of palpal tibia length, with a more-or-less finger-like apex; a very minute, tooth-shaped dorsal apophysis; cymbial spur two times shorter than tibia, beak-shaped; cymbial fold strongly developed and conspicuous in ventral and retrolateral views for approximately 2/3 of cymbium length; tip of cymbium long, ca. 1/2 of cymbium length. Tegulum oval, 1.6 times longer than wide; median apophysis long and filamentous, more than 1/2 of tegulum length, with a curved tip resembling a sickle in ventral view; embolus filiform, originating on the retrolateral flank (approximately 1 o’clock on tegulum), surrounding base and ending at conductor apex, its tip curved behind conductor and extending to apex of cymbium; conductor large, shaped like a blade point, base wide, gradually tapering toward apex.

**Female.** Distinctly larger and darker than male. Paratype (Fig. 2G, H) measured: TL 6.32; CL 2.40, CW 1.67, CI (CL/CW) 1.44; AL 3.92, AW 2.42. Eye diameters and interdistances: OAL 0.41, OAW 1.07; AME 0.11, ALE 0.13, PME 0.12, PLE 0.13; AME–AME 0.09, AME–ALE 0.15, PME–PME 0.19, PME–PLE 0.22; MOQA 0.39, MOQP 0.46, CLL 0.08. PMT: RMT = 3:3. STL 1.20, STW 1.06. Legs yellowish white, without distinct markings. Leg measurements: I 21.08 (5.36, 6.70, 6.72, 2.30), II 11.50 (3.11, 3.83, 3.66, 0.91), III 8.58 (2.35, 2.57, 2.85, 0.82), IV 12.28 (3.24 3.76, 4.27, 1.02); LL/CL 9.80.

Epigyne (Figs 2A–D). Atrium ca. two times wider than long, atrial anterior margin eyebrow-shaped and heavily sclerotized, posterior and lateral margins inconspicuous; receptacles are faintly visible through epigynal plate in ventral view; two copulatory openings, large and conjoined, located at posterior portion of epigynal plate; transparent copulatory ducts coiled, forming four entwined loops (including three ascending coils and one descending coil); receptacle reniform, separated by diameter of receptacle.

##### Distribution.

Known only from the type locality, Xishuangbanna, Yunnan, China.

#### Cheiracanthium duanbi

Taxon classificationAnimaliaAraneaeMiturgidae

Yu & Li sp. nov.

D8FCB224-E52D-58E9-AD41-8A9782E1994E

http://zoobank.org/0BC67D41-E295-4A3E-B8DB-BB84448A7B9C

**Figure 4.**
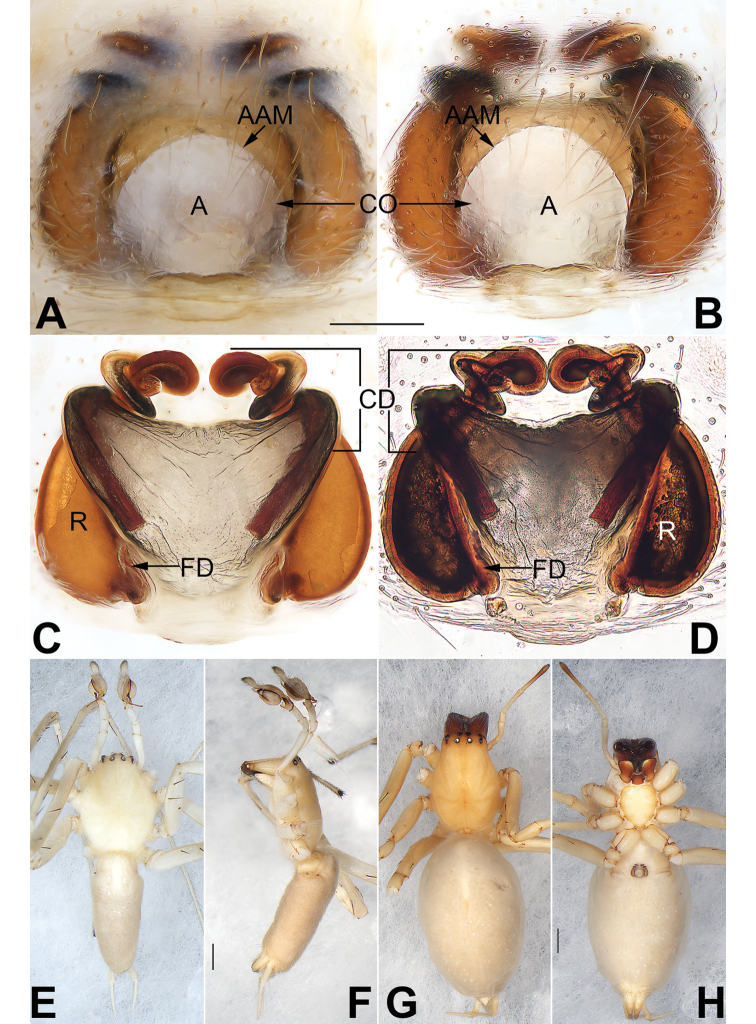
*Cheiracanthium duanbi* sp. nov., female paratype and male holotype. **A** epigyne, intact, ventral view **B** epigyne, cleared, ventral view **C** vulva, cleared, dorsal view **D** vulva, cleared and embedded in Arabic gum, dorsal view **E** male habitus, dorsal view **F** male habitus, lateral view **G** female habitus, dorsal view **H** female habitus, ventral view. Abbreviations: A = atrium; AAM = atrial anterior margin; CD = copulatory duct; CO = copulatory opening; FD = fertilization duct; R = receptacle. Scale bars: 0.2 mm (**A–D**); 1 mm (**E–H**).

Figures 3 , 4 , 7B , 8B , 9B , 10D–F

##### Holotype.

♂ (IZCAS-Ar 34743, YHCH030), China, Yunnan Province, Xishuangbanna, Mengla County, Menglun Town, Xishuangbanna Tropical Botanical Garden, G213 roadside, *Anogeissus acuminata* plantation, 21°53.748'N, 101°17.084'E, elevation ca. 620 m, 1.V.2019, Zhigang Chen leg. Paratype: 1♀ (IZCAS-Ar 34744, YHCH029), Xishuangbanna Tropical Botanical Garden, 21°54.007'N, 101°16.395'E, elevation ca. 620 m, 10.V.2019, Zilong Bai leg.

##### Etymology.

The specific name is derived from the Chinese pinyin ‘duǎn bì’, which means ‘short dagger’, and refers to the dagger-shaped retrolateral tibial apophysis; noun in apposition.

##### Diagnosis.

The male of *C. duanbi* sp. nov. can be distinguished from all other *Cheiracanthium* species, except *C. exquestitum* (Zhang and Yin 1999: 287, figs 8, 9), by having a distally wide cymbial spur, a spine-like median apophysis, and a beak-shaped conductor but can be distinguished from *C. exquestitum* by having: (1) the distal tip of the cymbial spur partly membranous and not forked (Figs 3A–C, 7B, 8B); with a sclerotized and forked apex in *C. exquestitum*; (2) the retrolateral tibial apophysis erect, like a short dagger, in retrolateral view (Figs 3C, 8B), instead of sinuate and hook-shaped as in *C. exquestitum*; (3) the median apophysis shorter (Figs 3B, C, 7B, 8B, 10D–F). The females are similar to those of *C. exquestitum* (Zhang and Yin 1999: 287, figs 10, 11), *C. rehobothense* Strand, 1915 (Bayer 2014: 29, figs 8a–c, 9a, b), *C. gratum* Kulczyński, 1897 (Merkens and Wunderlich 2000: 42, figs 6–9), and *C. fulvotestaceum* Simon, 1878 (Hänggi and Stäubli 2012: 64, fig. 5) by the general shape of the atrium and vulva but can be distinguished by the copulatory ducts having three turns (vs. copulatory ducts with four turns in *C. exquestitum*, with two turns in *C. rehobothense* and *C. gratum*) and by the larger atrium (lateral margin of atrium close to the receptacle in *C. duanbi* sp. nov. vs. distant from the receptacle in *C. fulvotestaceum*).

##### Description.

**Male.** Holotype (Figs 4E, F): TL 7.68; CL 3.64, CW 2.83, CI (CL/CW) 1.29; AL 4.01, AW 1.99. Carapace white, uniformly coloured, without any pattern. Eyes: in dorsal view, both anterior and posterior eye rows recurved, PER slightly wider than AER. All eyes dark, on tubercles. Eye sizes and interdistances: OAL 0.41, OAW 1.27; AME 0.18, ALE 0.20, PME 0.17, PLE 0.12; AME–AME 0.15, AME–ALE 0.22, PME–PME 0.28, PME–PLE 0.32; MOQA 0.51, MOQP 0.56, CLL 0.04. Chelicerae robust and coloured as carapace, both margins with two teeth. Sternum yellowish white, STL 1.87, STW 1.41. Labium and endites coloured as carapace. Legs white with greyish metatarsi and tarsi. Leg measurements: I 29.59 (7.23, 9.39, 9.69, 2.28), II 21.05 (5.77, 6.86, 6.58, 1.84), III 15.11 (3.81, 4.86, 5.08, 1.35), IV 20.96 (5.31, 6.81, 7.07, 1.77); LL/CL 8.13. Abdomen lanceolate, dorsally grey, lighter anteriorly, darker posteriorly; dorsum with a lengthwise white heart mark, 1/3 of opisthosoma length; venter greyish without any pattern.

Palp (Figs 3A–C, 7B, 8B, 9B, 10D–F). Tibia relatively long, ca. 2/3 of cymbium length, with two apophyses: a short and sclerotized retrolateral one, ca. 1/2 of palpal tibia length, with wide base and narrow apex, dagger-shaped; and a small, round, thumb-like prolateral apophysis; cymbial spur approximately as long as tibia, distal tip wide and partly membranous; cymbial fold well-developed and clearly visible in ventral and retrolateral views for ca. 1/2 length of cymbium; tip of cymbium long, ca. 1/2 of cymbium length. Tegulum oval, 1.4 times longer than wide, surface wrinkled; median apophysis small and hyaline, spine-like; embolus filiform, arising at approximately 12 o’clock position, terminating at approximately 11 o’clock position, tip covered by conductor; conductor short, wide, beak-shaped, base covering embolic base, tip covering embolar apex.

**Female.** Distinctly larger and darker than male. Paratype (Figs 4G, H): TL 10.32; CL 3.64, CW 2.79, CI (CL/CW) 1.30; AL 6.69, AW 4.20. Eye diameters and interdistances: OAL 53, OAW 1.46; AME 0.21, ALE 0.23, PME 0.18, PLE 0.17; AME–AME 0.12, AME–ALE 0.31, PME–PME 0.37, PME–PLE 0.41; MOQA 0.55, MOQP 0.74, CLL 0.06. PMT: RMT = 2:3. STL 1.79, STW 1.50. Legs yellowish brown, without distinct markings. Leg measurements: I 20.68 (5.27, 7.07, 6.06, 2.28), II 14.99 (4.25, 5.33, 4.00, 1.42), III 10.55 (2.91, 3.47, 2.97, 1.21), IV – (3.86, –, –, –); LL/CL 5.68.

Epigyne (Fig. 4A–D). Atrium large, located at posterior portion of epigynal plate, with delimited margin anteriorly and laterally, length is almost equivalent to width; receptacles and copulatory ducts prominent through epigynal plate in ventral view; two copulatory openings located at basolateral atrial borders; transparent copulatory ducts coiled, with three turns (including two ascending coils and one descending coil); receptacles elongated and pyriform, ca. two times longer than wide, separated by 1.5 diameters.

##### Distribution.

Known only from the type locality, Xishuangbanna, Yunnan, China.

#### Cheiracanthium gou

Taxon classificationAnimaliaAraneaeMiturgidae

Yu & Li sp. nov.

5D81B754-419D-5DCF-B5A7-CAA1EB73BDC2

http://zoobank.org/BC42EBA3-7A0D-44FE-9274-5E5EB8FDB88D

**Figure 5.**
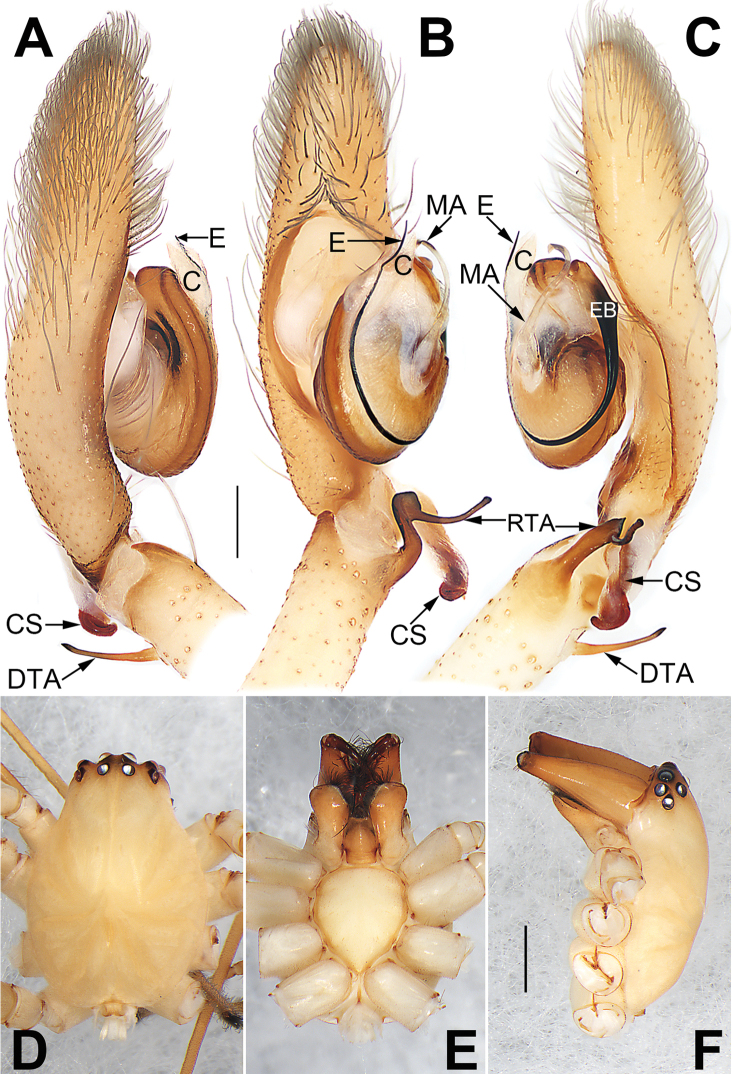
*Cheiracanthium gou* sp. nov., male holotype. **A** Flipped right palp, prolateral view **B** left palp, ventral view **C** left palp, retrolateral view **D** habitus, dorsal view **E** habitus, ventral view **F** habitus, lateral view. Abbreviations: C = conductor; CS = cymbial spur; DTA = dorsal tibial apophysis; E = embolus; EB = embolic base; MA = median apophysis; RTA = retrolateral tibial apophysis. Scale bars: 0.2 mm (**A–C**); 1 mm (**D–F**).

Figures 5 , 7C , 8C , 9C , 10G–I

##### Holotype.

♂ (IZCAS-Ar 34746, YHCH016), China, Yunnan Province, Xishuangbanna, Mengla County, Menglun Town, Xishuangbanna Tropical Botanical Garden, 48 km landmark in the reserve, seasonal rainforest, 21°58.704'N, 101°19.748'E, elevation ca. 1088 m, 12.VIII.2011, Guo Zheng leg.

##### Etymology.

The specific name is derived from the Chinese pinyin ‘gōu’, which means ‘hook’, and refers to the curved distal tip of the cymbial spur which is shaped like a hook; noun in apposition.

##### Diagnosis.

Males of this new species can be easily distinguished from all other *Cheiracanthium* species by the structure of the palp. The retrolateral tibial apophysis consists of a thin distal half and a wide basal half. The cymbial spur is partly membranous proximally and sclerotized distally with the distal tip blunt and thick, hook-shaped (Figs 5A–C, 7C, 8C, 9C). By contrast, in almost all known *Cheiracanthium* species, the retrolateral tibial apophysis and the cymbial spur cannot be easily divided into two parts, and the distal tip of the cymbial spur is usually sharply pointed, such as in *C. daofeng* sp. nov. and *C. duanbi* sp. nov. (Figs 1A–C, 3A–C, 7A, B, 8A, B, 9A, B).

##### Description.

**Figure 6.**
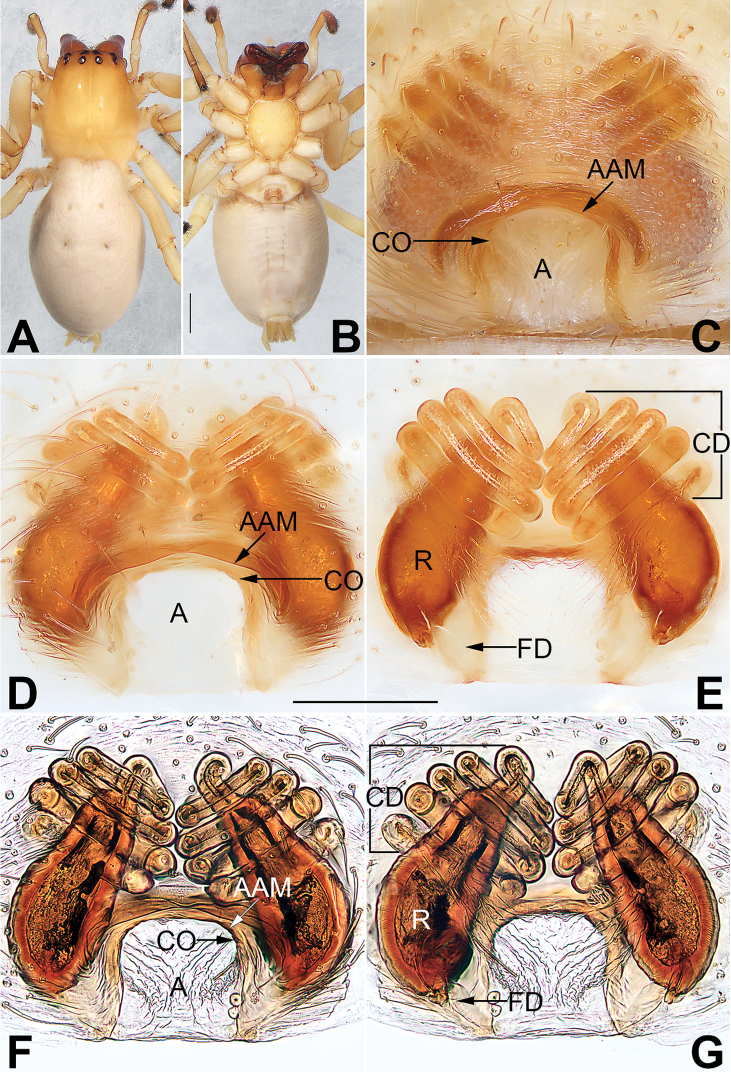
*Cheiracanthium wuquan* sp. nov., female holotype. **A** Habitus, dorsal view **B** habitus, ventral view **C** epigyne, intact, ventral view **D** epigyne, cleared, ventral view **E** vulva, cleared, dorsal view **F** epigyne, cleared and embedded in Arabic gum, ventral view **G** vulva, cleared and embedded in Arabic gum, dorsal view. Abbreviations: A = atrium; AAM = atrial anterior margin; CD = copulatory duct; CO = copulatory opening; FD = fertilization duct; R = receptacle. Scale bars: 1 mm (**A, B**); 0.2 mm (**C–G**).

**Male.** Holotype (Fig. 6D–F): TL –; CL 3.63, CW 2.83, CI (CL/CW) 1.28; AL –, AW –. Carapace pale yellow, uniformly coloured, without distinct pattern; cephalic region inconspicuously raised, cervical groove and radial grooves distinct, tegument smooth, clothed with short, fine hairs. Eyes: in dorsal view, both anterior and posterior eye rows recurved, PER slightly wider than AER. All eyes dark, on tubercles. Eye sizes and interdistances: OAL 0.49, OAW 1.32; AME 0.22, ALE 0.20, PME 0.17, PLE 0.18; AME–AME 0.17, AME–ALE 0.18, PME–PME 0.22, PME–PLE 0.28; MOQA 0.53, MOQP 0.58, CLL 0.09. Chelicerae with three teeth on promargin and three on retromargin, with long red fangs. Sternum pale yellow, STL 1.67, STW 1.32. Labium and endites orange. Legs distinctly long, yellowish white, with brown metatarsi and tarsi, without distinct markings. Leg measurements: I 32.75 (8.38, 1.062, 11.29, 2.46), II – (5.32, –, –, –), III missing, IV 22.07 (6.4, 6.34, 7.72, 1.61); LL/CL 9.02. Abdomen missing.

**Figure 7.**
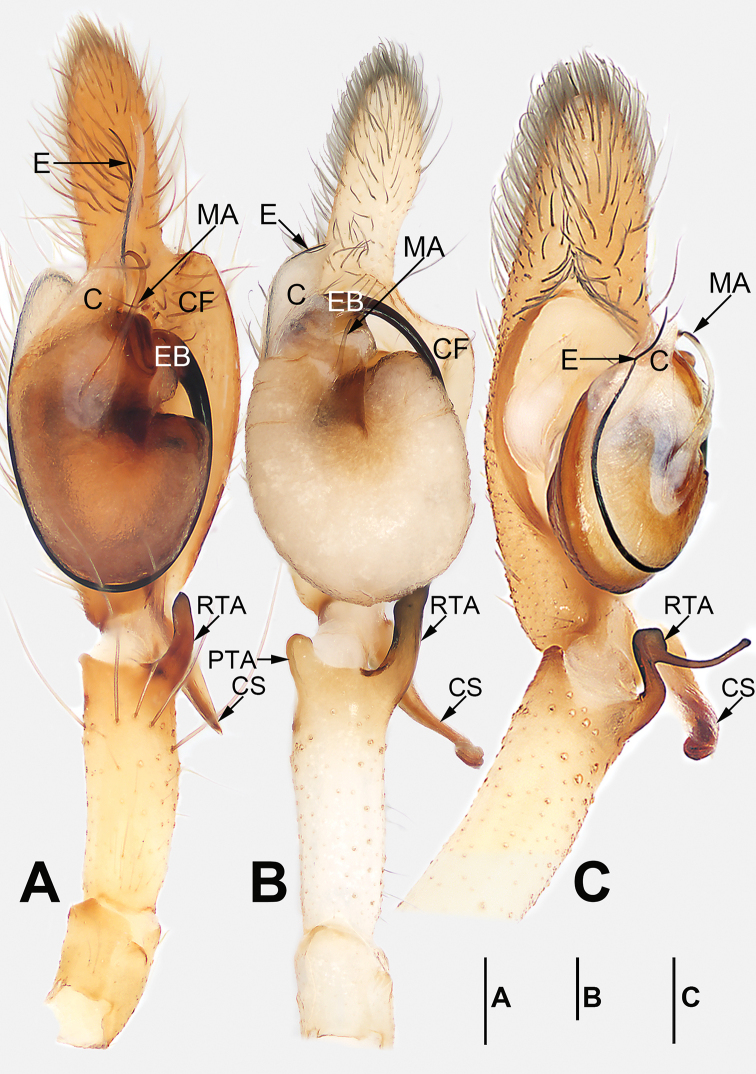
*Cheiracanthium* spp., left palp, ventral view. **A***C. daofeng* sp. nov., male holotype **B***C. duanbi* sp. nov., male holotype **C***C. gou* sp. nov., male holotype. Abbreviations: C = conductor; CF = cymbial fold; CS = cymbial spur; E = embolus; EB = embolic base; MA = median apophysis; RTA = prolateral tibial apophysis; RTA = retrolateral tibial apophysis. Scale bars: 0.2 mm.

**Figure 8.**
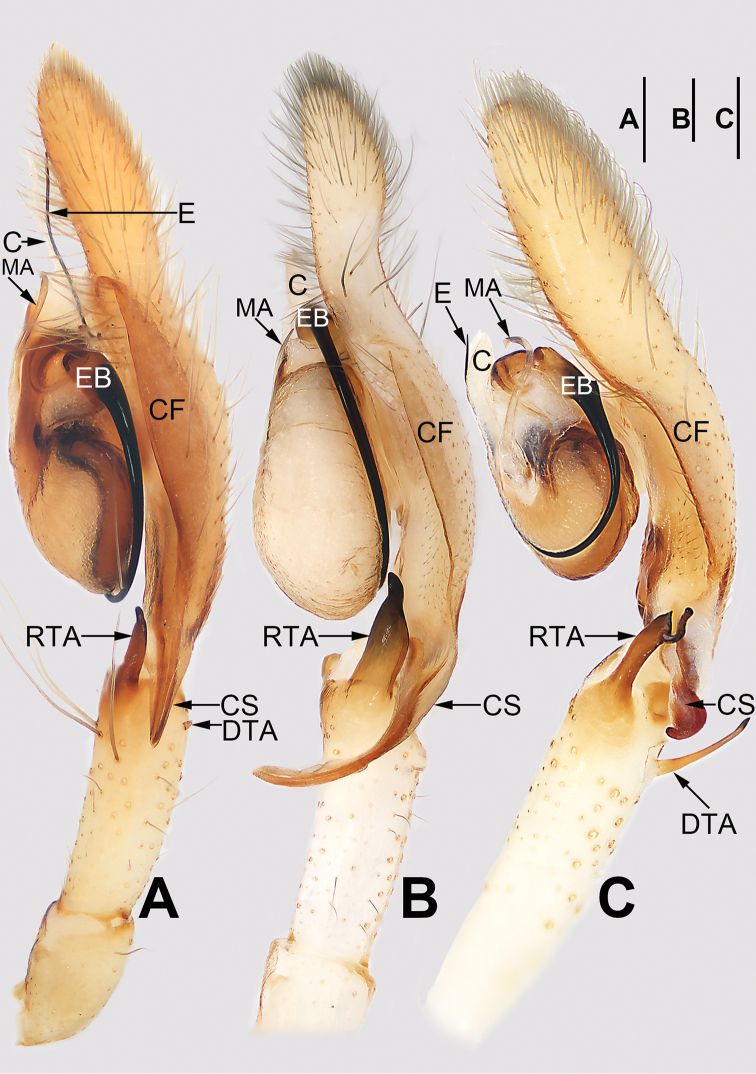
*Cheiracanthium* spp., left palp, retrolateral view. **A***C. daofeng* sp. nov., male holotype **B***C. duanbi* sp. nov., male holotype **C***C. gou* sp. nov., male holotype. Abbreviations: C = conductor; CF = cymbial fold; CS = cymbial spur; DTA = dorsal tibial apophysis; E = embolus; EB = embolic base; MA = median apophysis; RTA = retrolateral tibial apophysis. Scale bars: 0.2 mm.

**Figure 9.**
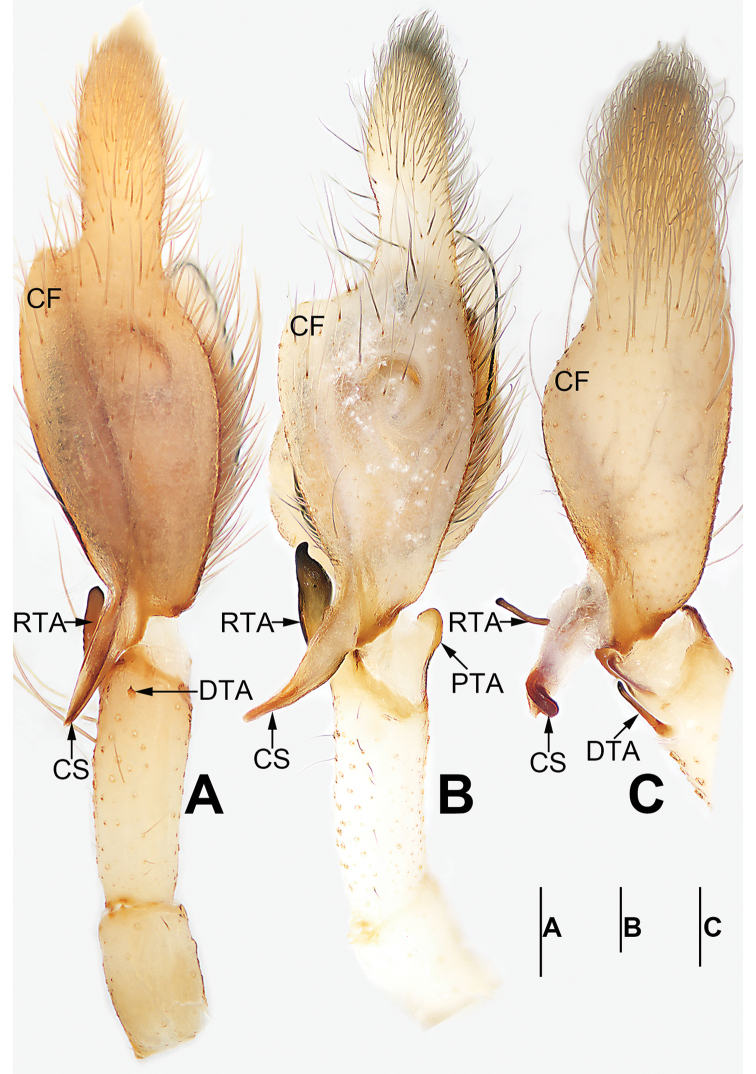
*Cheiracanthium* spp., left palp, dorsal view. **A***C. daofeng* sp. nov., male holotype **B***C. duanbi* sp. nov., male holotype **C***C. gou* sp. nov., male holotype. Abbreviations: CF = cymbial fold; CS = cymbial spur; DTA = dorsal tibial apophysis; PTA = prolateral tibial apophysis; RTA = retrolateral tibial apophysis. Scale bars: 0.2 mm.

**Figure 10.**
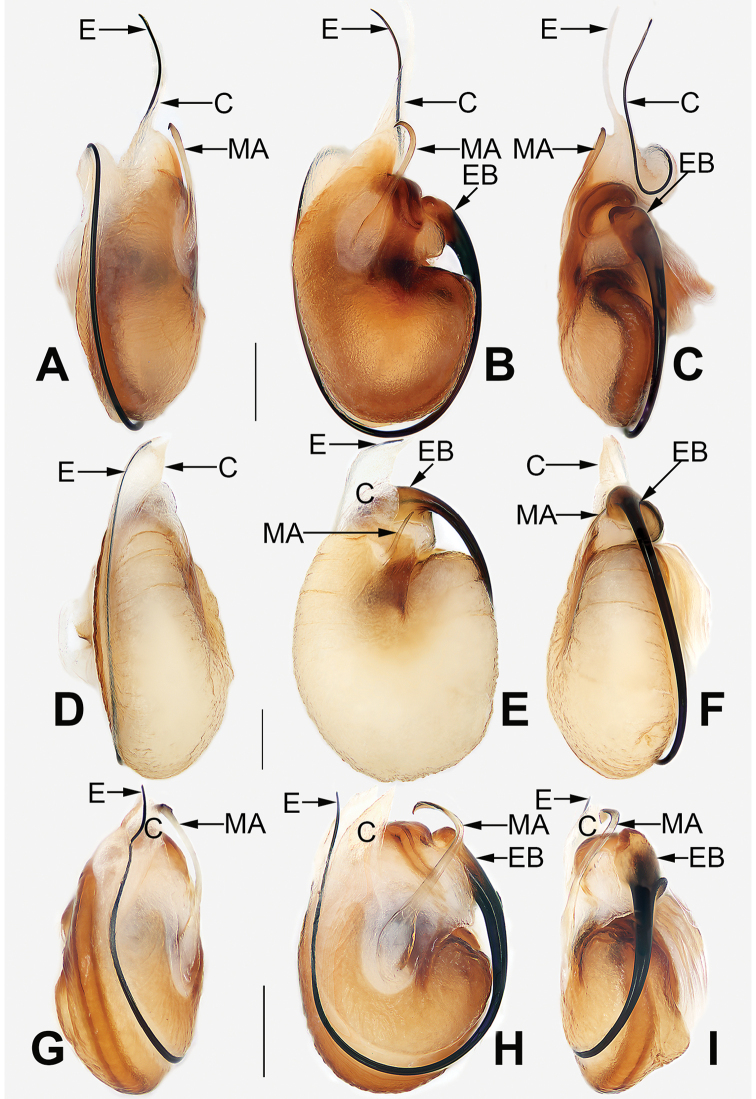
*Cheiracanthium* spp., left bulb, prolateral view (**A, D, G**), ventral view (**B, E, H**), retrolateral view (**C, F, I**).**A–C***C. daofeng* sp. nov. **D–F***C. duanbi* sp. nov. **G–I***C. gou* sp. nov. Abbreviations: C = conductor; E = embolus; EB = embolic base; MA = median apophysis. Scale bars: 0.2 mm.

Palp (Figs 5A–C, 7C, 8C, 9C, 10G–I). Tibia with two apophyses: long and sclerotized retrolateral apophysis, ca. 1/3 of palpal tibia length, with thin distal half and wide basal half; and a short, thin, stalk-like dorsal apophysis; cymbial spur short, ca. 1/3 of cymbium length, partly membranous proximally, heavily sclerotized distally, distal tip curved and blunt; cymbial fold poorly developed and indistinct in ventral and retrolateral views for ca. 1/2 the length of cymbium; tip of cymbium long, ca. 1/2 of cymbium length. Bulb elongated, 1.5 times longer than wide; median apophysis long and hyaline, more than 1/2 of tegulum length, with wide base, thin middle part, and hook-shaped tip; embolus originates at ca. 1 o’clock position, surrounds base, and ends atop conductor at distal end of tegulum; conductor short, thick, membranous.

**Female.** Unknown.

##### Comments.

According to the WSC (2020), a total of ten *Cheiracanthium* species from China are known only from females: *C. approximatum* O. P.-Cambridge, 1885, *C. escaladae* Barrion et al., 2013, *C. fujianense* Gong, 1983, *C. hypocyrtum* Zhang & Zhu, 1993, *C. liuyangense* Xie et al., 1996, *C. olliforme* Zhang & Zhu, 1993, *C. potanini* Schenkel, 1963, *C. solidum* Zhang et al., 1993, *C. sphaericum* Zhang et al., 1993, and *C. longtailen* Xu, 1993. Among them, *C. escaladae* is supposedly a *Clubiona* species based on epigyne morphology, *C. approximatum* and *C. potanini* are doubtful or invalid species because of the poor original illustrations and descriptions, *C. liuyangense* may be a synonym of *C. taegense* Paik, 1990, and *C. longtailen* is considered a junior synonym of *C. pichoni* Schenkel, 1963. The remaining five species can be tentatively considered valid species. In addition, *C. spectabile* (Thorell, 1887) from Myanmar is known by the male but is not illustrated. We cannot rule out the possibility that the above six species are conspecific to *C. gou* sp. nov.

##### Distribution.

Known only from the type locality, Xishuangbanna, Yunnan, China.

#### Cheiracanthium wuquan

Taxon classificationAnimaliaAraneaeMiturgidae

Yu & Li sp. nov.

4DDCCB48-74A1-5B24-88E2-664A376BEDED

http://zoobank.org/6BEB7F25-F29A-47A9-BFAB-E5D11EDECC36

Figure 6

##### Holotype.

♀ (IZCAS-Ar 34747, YHCH020), China, Yunnan Province, Xishuangbanna, Mengla County, Menglun Town, Xishuangbanna Tropical Botanical Garden, 48 km landmark in the reserve, seasonal rainforest; 21°58.704'N, 101°19.748'E, elevation ca. 1088 m, 12.VIII.2011, Guo Zheng leg.

##### Etymology.

The specific name is derived from the Chinese pinyin ‘wǔ quān’, which means ‘five loops’, and refers to the coiled copulatory ducts, forming five entwined coils; noun in apposition.

##### Diagnosis.

The female of the new species is similar to those of *C. japonicum* Bösenberg & Strand, 1906 (Zhu and Zhang 2011: 341, fig. 246A, B), *C. simaoense* Zhang & Yin, 1999 (Zhang and Yin 1999: 286, figs 6, 7), *C. turiae* Strand, 1917 (Deeleman-Reinhold 2001: 234, figs 292, 293), and *C. virescens* (Sundevall, 1833) (Zhu and Zhang 2011: 346, fig. 250A, B) by the general shape of the atrium and receptacles and the coiling of the copulatory duct around the distal part of the receptacle. The new species can be easily distinguished by the different number of copulatory duct coils (copulatory ducts with five turns in *C. wuquan* sp. nov. vs. three turns in *C. simaoense* and *C. virescens* and four turns in *C. japonicum* and *C. turiae*) (Figs 6C–G).

##### Description.

**Female.** Holotype (Fig. 6A, B): TL 6.85; CL 2.69, CW 2.42, CI (CL/CW) 1.11; AL 4.15, AW 2.78. Carapace yellowish orange except yellowish brown ocular area, without distinct pattern; cervical groove and radial grooves indistinct. Eyes: both anterior and posterior eye rows almost straight in dorsal view, PER slightly wider than AER. All eyes dark, with black rings. Eye sizes and interdistances: OAL 0.37, OAW 1.10; AME 0.15, ALE 0.11, PME 0.14, PLE 0.14; AME–AME 0.20, AME–ALE 0.23, PME–PME 0.30, PME–PLE 0.33; MOQA 0.46, MOQP 0.57, CLL 0.04. Chelicerae robust and brownish red, with long, red wine-coloured fangs, with three teeth on promargin and two on retromargin. Sternum pale yellow, STL 1.35, STW 1.12. Labium and endites light orange. Legs yellowish white with brown metatarsi and tarsi, without distinct markings. Leg measurements: I 9.38 (2.54,3.35, 2.73, 0.77), II 7.83 (2.00, 2.88, 2.23, 0.72), III 5.78 (1.64, 1.86, 1.65, 0.62), IV 7.96 (2.41, 2.43, 2.42, 0.70); LL/CL 3.49. Abdomen oval, uniformly grey, dorsum with two pairs of conspicuous muscle depressions; venter medially with two longitudinal dotted lines.

Epigyne (Figs 6C–G). Atrium large, slightly wider than long, located at posterior portion of epigynal plate, with arched anterior hood and indistinct posterior margin; two copulatory openings, large and contiguous, located at basolateral atrial borders; receptacles and copulatory ducts conspicuous through epigynal plate in ventral view; transparent copulatory ducts coiled, with five ascending turns, connecting receptacle anteriorly; receptacles elongated and separated by 1.5 diameters, pear shaped, ducts coil around distal part.

**Male.** Unknown.

##### Comments.

Due to the large and long, ovoid receptacles and the slender copulatory duct encircling the anterior part of the receptacles, we justifiably place *C. wuquan* sp. nov. in *Cheiracanthium* sensu stricto. Until now, five *Cheiracanthium* species from China are known from males only: *C. antungense* Chen & Huang, 2012, *C. echinulatum* Zhang, Zhang & Yu, 2018, *C. gobi* Schmidt & Barensteiner, 2000, *C. ningmingense* Zhang & Yin, 1999, and *C. chayuense* Li & Zhang, 2019 (WSC 2020). Based on the palpal structure, with the exception of *C. ningmingense*, the other four species do not belong to *Cheiracanthium* sensu stricto (Wunderlich 2012). *C. ningmingense* is presently known from Ningming county in Guangxi (type locality), Shimen county in Hunan, and Xishuangbanna in Yunnan. However, *C. ningmingense* maybe a junior synonym of one known species.

##### Distribution.

Known only from the type locality, Xishuangbanna, Yunnan, China.

#### Sinocanthium

Taxon classificationAnimaliaAraneaeMiturgidae

Yu & Li gen. nov.

9B66D7FD-0A12-5B59-975F-F4442BF0EA45

http://zoobank.org/E11D612A-3AC7-4F35-BCAD-D0B2EAD27FCF

##### Type species.

*Sinocanthium shuangqiu* Yu & Li, sp. nov.

##### Etymology.

The generic name is derived from its similarity to *Cheiracanthium* and the Latin adjective Sino- for Chinese referring to the distribution region of the genus. The gender is neuter.

##### Diagnosis.

**Figure 11.**
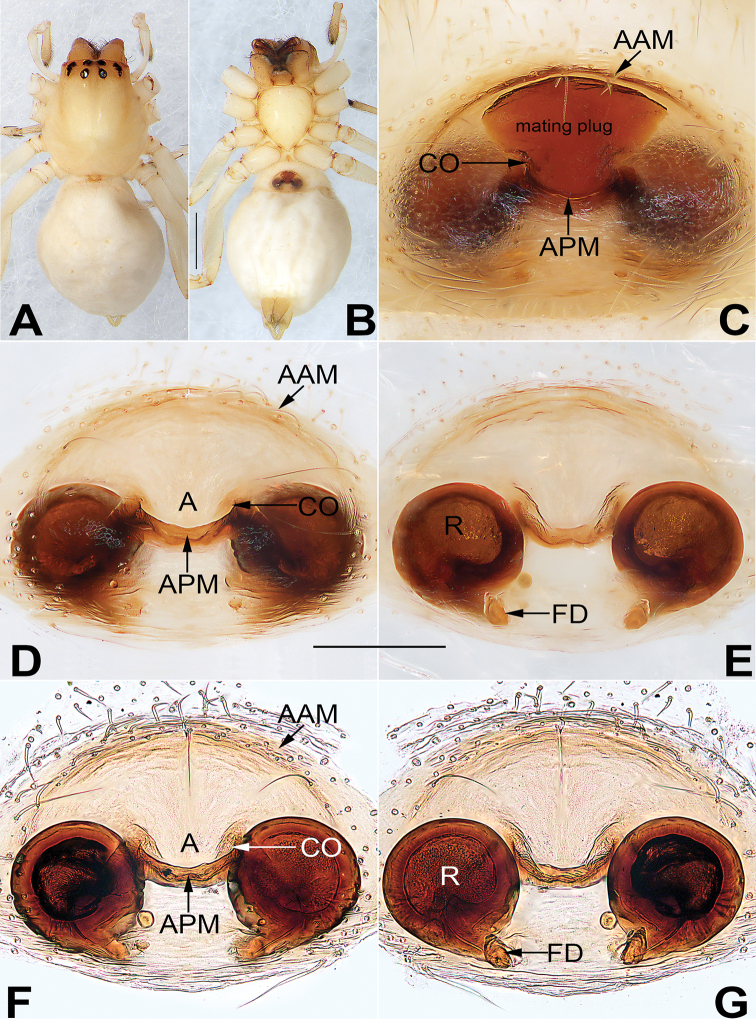
*Sinocanthium shuangqiu* sp. nov., female holotype. **A** habitus, dorsal view **B** habitus, ventral view **C** epigyne, intact, ventral view **D** epigyne, cleared, ventral view **E** vulva, cleared, dorsal view **F** epigyne, cleared and embedded in Arabic gum, ventral view **G** vulva, cleared and embedded in Arabic gum, dorsal view. Abbreviations: A = atrium; AAM = atrial anterior margin; APM = atrial posterior margin; CO = copulatory opening; FD = fertilization duct; R = receptacle. Scale bars: 1 mm (**A, B**); 0.2 mm (**C–G**).

*Sinocanthium* gen. nov. resembles *Cheiracanthium* by the similar habitus (Figs 11A, B, 4G, H, 6A, B) but is consistently separable by the shape of the epigyne. *Sinocanthium* gen. nov., as in most *Cheiracanthium* species, has a yellow body, a wide cephalic part, long legs, subequal eyes, and parallel eye rows of equal width occupying the greater width of the head. It can be distinguished from *Cheiracanthium* sensu lato by the absence of copulatory ducts (Fig. 11E, G) (vs. copulatory ducts with variable lengths but distinct in all *Cheiracanthium* species, including *C. daofeng* sp. nov., *C. duanbi* sp. nov., and *C. wuquan* sp. nov. (Figs 2C, D, 4C, D, 6E, G), and by the atrium located anteriorly with a rebordered posterior margin (Figs 11C, D, F) (vs. atrium located anteriorly or centrally, atrial hood located anteriorly in *Cheiracanthium*).

##### Description.

Same as for the species.

##### Composition.

Type species only.

##### Comments.

Based on the original figures and text descriptions of the epigynes, *Cheiracanthium* sensu lato can be further divided into at least four or five different taxa (Wunderlich 2012). The morphology of the epigyne exhibits very high diversity. However, all *Cheiracanthium* species related to the generotype have copulatory ducts, even though the shapes, lengths, and courses of the copulatory ducts are variable. Despite the variable shapes, the atria of the different taxa are located posteriorly or centrally and are usually rebordered anteriorly and laterally. Obviously, *Sinocanthium shuangqiu* sp. nov. cannot be placed in *Cheiracanthium* sensu lato because of the peculiar structure of the epigyne, so we described a new genus to accommodate it. Although we examined only one female of *S. shuangqiu* sp. nov., our specimen is strikingly different from all *Cheiracanthium* species: The atrium is located anteriorly, the atrial posterior margin is rebordered, and copulatory ducts are absent, supporting our decision.

#### Sinocanthium shuangqiu

Taxon classificationAnimaliaAraneaeMiturgidae

Yu & Li sp. nov.

F4922C2C-91AF-5CDB-8E21-0555C224B405

http://zoobank.org/C9C8FB70-E418-4B37-8EBF-EA5533BE4373

Figure 11

##### Holotype.

♀ (IZCAS-Ar 34745, YHCH011), China, Yunnan Province, Xishuangbanna, Mengla County, Menglun Town, Xishuangbanna Tropical Botanical Garden, 48 km landmark in the reserve, seasonal rainforest; 21°58.704'N, 101°19.748'E, elevation ca. 1088 m, 12.VIII.2011, Guo Zheng leg.

##### Etymology.

The specific name is derived from the Chinese pinyin ‘shuāng qiú ‘, which means ‘double ball’, and refers to the two bulb-shaped receptacles; noun in apposition.

##### Diagnosis.

The new species is easily distinguished from other cheiracanthiids by the epigyne, which has a fan-shaped fovea that is rebordered posteriorly and by the absence of copulatory ducts.

##### Description.

**Female.** Holotype (Fig. 11A, B): TL 5.02; CL 2.13, CW 1.71, CI (CL/CW) 1.25; AL 2.89, AW 2.06. Carapace pale yellow, ocular area brown, a pair of brown lateral bands originating from behind PME and PLE, almost reaching posterior half of carapace. Eyes: AER almost straight, PER wider than AER and slightly procurved in dorsal view. All eyes dark, with black rings. Eye sizes and interdistances: OAL 0.51, OAW 0.93; AME 0.16, ALE 0.16, PME 0.17, PLE 0.13; AME–AME 0.21, AME–ALE 0.26, PME–PME 0.28, PME–PLE 0.34; MOQA 0.49, MOQP 0.58, CLL 0.11. Chelicerae with orange base, fangs red wine-coloured, both margins without teeth. Sternum yellowish, STL 1.17, STW 0.94. Labium and endites coloured as ocular area. Legs yellowish white, without any markings. Leg measurements: I and II missing, III – (–, 1.56, 1.36, 0.57), IV 8.02 (2.35, 2.59, 2.37, 0.71). Abdomen (Fig. 11A, B) oval, dorsally yellowish white, dorsum with two pairs of inconspicuous muscle depressions; venter white with no distinct pattern.

Epigyne (Fig. 11C–G). Atrium large and fan-shaped, located anteriorly on epigynal plate, margin delimited, atrial anterior margin long and indistinct, posterior margin heavily sclerotized; atrium ca. two times wider than long, blocked by mating plug before cleaning; receptacles clearly visible through the tegument in ventral view; copulatory openings small, located at basolateral atrial borders; receptacles perfectly round, separated by 0.8 diameters.

**Male.** Unknown.

##### Distribution.

Known only from the type locality, Xishuangbanna, Yunnan, China.

## Supplementary Material

XML Treatment for Cheiracanthium

XML Treatment for Cheiracanthium daofeng

XML Treatment for Cheiracanthium duanbi

XML Treatment for Cheiracanthium gou

XML Treatment for Cheiracanthium wuquan

XML Treatment for Sinocanthium

XML Treatment for Sinocanthium shuangqiu
